# INCREASING THE VISIBILITY OF OLDER ADULTS IN RESEARCH: THE IMPORTANCE OF AGE, SEX, AND FRAILTY CONSIDERATIONS

**DOI:** 10.1093/geroni/igae098.1648

**Published:** 2024-12-31

**Authors:** Paula Rochon, Joyce Li

**Affiliations:** Women’s College Hospital, Toronto, Ontario, Canada; Women’s College Research Institute, Toronto, Ontario, Canada

## Abstract

Older adults are often considered as a single group with differences between women and men across age groups overlooked. Taking account of sex, age, and their intersection throughout the research process is crucial in minimizing inequities, and to improve the health and wellbeing of the aging population. Age and sex disaggregated data are data separated and analyzed by female, male and age categories, revealing important differences between the sexes and across the life course. This is often missing in research and can result in the oversimplification of study results. As men and women age, they can experience different health conditions, and levels of frailty, leading to the need for tailored treatments. One illustration of this is exploring the burden of disease in Canada, where disaggregating data by sex and age makes key differences in many diseases impacting older adults visible, that may otherwise be missed (for example, heart disease and dementia). Age, sex, and frailty considerations are important throughout the entire continuum of the research process, from question development to dissemination of results to ensure they are accounted for. This presentation will explore why incorporating sex, age, frailty and their intersection are important in health research, how this consideration adds value, and applications of this approach in practice to increase the visibility of older women and men in research, contributing to the ‘fortitude factor’.

